# Inherited SHQ1 mutations impair interaction with NAP57/dyskerin, a major target in dyskeratosis congenita

**DOI:** 10.1002/mgg3.314

**Published:** 2017-08-15

**Authors:** Jonathan Bizarro, U. Thomas Meier

**Affiliations:** ^1^ Department of Anatomy and Structural Biology Albert Einstein College of Medicine 1300 Morris Park Avenue Bronx New York 10461

**Keywords:** compound heterozygous mutations, dyskeratosis congenita, H/ACA RNP, Hoyeraal‐Hreidarsson syndrome, protein‐protein interaction, ribonucleoprotein (RNP) biogenesis

## Abstract

**Background:**

The inherited bone marrow failure syndrome dyskeratosis congenita (DC) is most frequently caused by mutations in *DKC1* (MIM# 300126), the gene encoding NAP57 (aka dyskerin). The typically missense mutations modulate the interaction of NAP57 with its chaperone SHQ1, but no DC mutations have been identified in *SHQ1* (MIM# 613663). Here, we report on two compound heterozygous mutations in *SHQ1* in a patient with a severe neurological disorder including cerebellar degeneration.

**Methods:**

The SHQ1 mutations were identified by patient exome sequencing. The impact of the mutations was assessed in pulldown assays with recombinant NAP57.

**Results:**

The SHQ1 mutations were the only set of mutations consistent with an autosomal recessive mode of inheritance. The mutations map to the SHQ1‐NAP57 interface and impair the interaction of the recombinant SHQ1 variants with NAP57.

**Conclusion:**

Intrauterine growth retardation and the neurological phenotype of the patient are reminiscent of the severe clinical variant of DC, the Hoyeraal‐Hreidarsson syndrome (HH). Hence, *SHQ1* screening may be warranted in patients with inherited bone marrow failure syndromes.

H/ACA ribonucleoproteins (RNPs) consist of a large family of ~135 nucleotide‐long H/ACA RNAs including telomerase RNA and two sets of four core proteins, NAP57 (aka dyskerin), NOP10, NHP2, and GAR1 (Yu and Meier 2014). Their functions are important for ribosome biogenesis, translation regulation, pre‐mRNA splicing, and telomere maintenance. Many components and assembly factors of H/ACA RNPs are targets of the inherited bone marrow failure syndrome dyskeratosis congenita (DC), including NAP57, NOP10, NHP2, telomerase RNA and reverse transcriptase, and the maturation factors nuclear assembly factor 1 (NAF1), poly(A)‐specific ribonuclease (PARN), and Cajal body localizing factor (Wdr79/TCAB1) (Bertuch 2016; Wegman‐Ostrosky and Savage 2017). Although telomerase is only one of over 500 different H/ACA RNPs, critically short telomeres are a hallmark of all DC patients (Alter et al. 2012). The predominant target of DC and its clinically most severe variant Hoyeraal‐Hreidarsson syndrome (HH) is the major H/ACA RNP core protein NAP57 (Heiss et al. 1998). Despite many of the NAP57 variants affecting the interaction with its chaperone and H/ACA RNP assembly factor SHQ1, no DC mutations have been identified in SHQ1.

We report on a patient with intrauterine growth retardation and a severe neurological disorder including cerebellar degeneration. The patient was born at gestational week 29 by urgent cesarean section due to intrauterine growth retardation and sparse fetal movement. Birth weight was 1000 grams. Significant feeding difficulties and lack of acquisition of developmental milestones were noted since birth. Severe epileptic disorder was first noted at 2 years of age. Brain MRI, performed at 1 year of age revealed cerebellar hypoplasia, ventricular dilatation and marked atrophy. Blood counts were normal and no mucocutaneous findings were reported. Presently he is in a vegetative state with cortical atrophy and spastic quadriplegia.

Exome analysis of the patient DNA was performed on exon targets captured using SureSelect Human All Exon 50 Mb Kit V.4 (Agilent Technologies, Santa Clara, California, USA). Sequences were determined by HiSeq2000 (Illumina, San Diego, CA, USA). The full sequencing methodology and variant interpretation protocol were previously described (Ta‐Shma et al. 2017). Following reads alignment to human genome (hg19), variant calling and filtration, all remaining variants were heterozygous, and there were no variants in any of the genes of the Clinical Genomic Database (CGD) that could fit an X‐linked or autosomal recessive or dominant mode of inheritance (Solomon et al. 2013). Among the variants residing in non‐CGD genes, we noted two variants in the same gene. These were p.R335C (c.1003C>T) and p.A426V (c.1277C>T) (Fig. 1A, NM_018130.2), in the H/ACA RNP assembly factor and NAP57 chaperone SHQ1 (NP_060600.2). Sanger sequencing of his parents and two healthy siblings confirmed segregation of the mutations with the disease state in this family (Fig. 1A).

**Figure 1:**
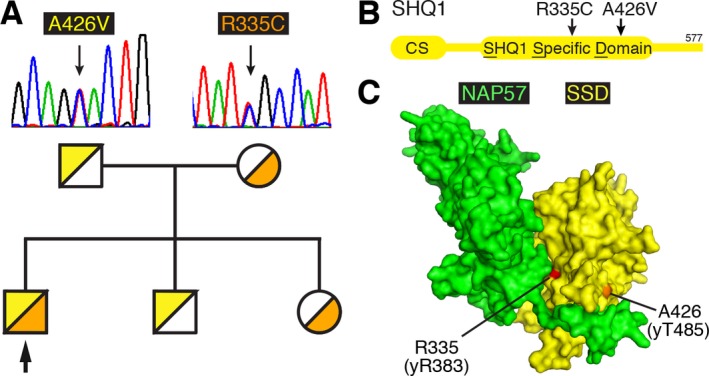
Pedigree and location of SHQ1 mutations. (A) Sanger sequencing of the father and mother confirms the hemizygous mutations of p.A426V (c.1277C>T) and p.R335C (c.1003C>T) respectively (NM_018130.2). The pedigree of the family is indicated below with the proband pinpointed by an arrow. (B) Schematic of the SHQ1 protein sequence with the N‐terminal CS and the central SHQ1 specific domain (SSD) that harbors the mutations. (C) Location of the equivalent positions mapped onto the structure of the yeast SSD – NAP57 (Cbf5p) complex (PDB ID: 3uai).

Both compound heterozygous mutations lie in the heart of the unique SHQ1 specific domain (SSD) of SHQ1 that interacts with NAP57 through many of the same contacts that are eventually replaced by an H/ACA RNA (Fig. 1B) (Li et al. 2011; Walbott et al. 2011). To identify the location of the R335C and A426V mutations within the SHQ1‐NAP57 complex, we mapped the equivalent positions on the structure of the SSD of yeast SHQ1 (Fig. 1C). Whereas R335 is conserved in yeast SHQ1, yR385, the position of A426 is closest to yT485 based on the alignment of 90 SHQ1 proteins (Li et al. 2011). Both amino acids in the yeast protein are solvent accessible but are buried by at least 10 Å^2^ when complexed with yeast NAP57, Cbf5p (Fig. 1C). Therefore, we tested if mutation of these amino acids impaired interaction of the recombinant human proteins.

To assess differences in binding between wild type and mutant proteins more sensitively, we studied the interaction with NAP57 of the SSD alone, which binds less rigorously in the absence of the clamp forming CS domain of SHQ1 (Machado‐Pinilla et al. 2012). Indeed, in our amylose resin pulldown experiments of maltose binding protein (MBP) fused to NAP57, binding of the individual SSD mutants R335C and A426V was reduced to 54% and 79% of wild type SSD, respectively (*P *<* *0.0001; Fig. 2). When mixing equal amounts of both mutants in an attempt to replicate in vitro a compound heterozygous situation, binding was reduced to 59% (*P *<* *0.0001; Fig. 2). Interestingly, the more extreme amino acid transition of yR385 to glutamate (rather than cysteine as in the patient) almost completely abolished binding and barely supported growth above 20°C when replacing the essential Shq1p in yeast (Li et al. 2011). Thus, the two SHQ1 patient mutations impair the interaction of SHQ1 with NAP57 similarly to DC mutations in NAP57 (Grozdanov et al. 2009; Machado‐Pinilla et al. 2012).

**Figure 2:**
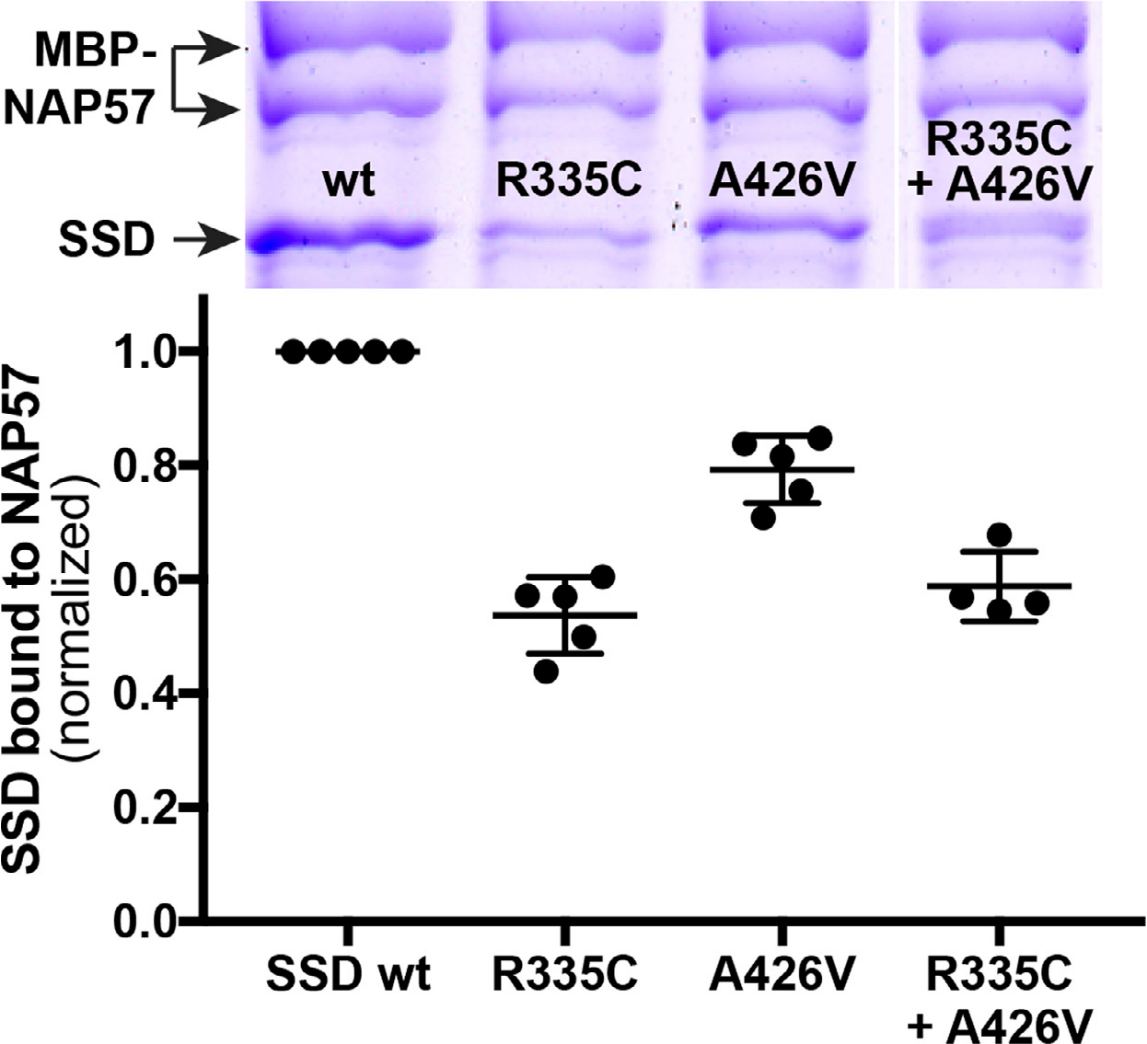
Pulldowns of recombinant wild type (wt) and mutant SSD with full‐length NAP57 fused to MBP using amylose beads. (Above) Representative coomassie blue stained denaturing polyacrylamide gel with the bands labeled on the left. Note the MBP‐NAP57 migrates as a doublet. (Below) Quantification of the SSD bands relative to MBP‐NAP57. Between 4 and 5 independent experiments were normalized to wild type SSD. In the last lane, the two variant proteins were mixed 1:1.

SHQ1 could be another gene affected in DC and the seventh in the pathway of H/ACA RNP maturation including the core proteins and telomerase reverse transcriptase and RNA. Biallelic transmission may be the only tolerable manner to inherit SHQ1 mutations, which otherwise might be fatal. In fact, DC causing mutations in other genes are transmitted in the same manner. For example, biallelic mutations in the H/ACA RNA Cajal body localizing factor Wdr79/TCAB1 and in the H/ACA RNA maturation factor poly(A)‐specific ribonuclease PARN have been identified in DC patients (Zhong et al. 2011; Dhanraj et al. 2015; Tummala et al. 2015).

Although the patient symptoms are reminiscent of the most severe clinical variant of DC, HH, we cannot be certain that they are caused by the SHQ1 variants, which could constitute very rare polymorphisms. In fact, R335C (rs746829352:A>G) is one of 388 single nucleotide polymorphisms (SNPs) reported in the NCBI dbSNP database for the SHQ1 coding region, but no SNP is reported for the A426 codon. Nevertheless, the R335C allele is extremely rare with only four counts in the ~60,000 exomes of the Exome Aggregation Consortium (A = 0.00003; ExAC, Cambridge, MA) (Lek et al. 2016). In contrast, the A426V allele has never been observed thus far, rendering the likelihood of the two variants co‐occurring by chance exceedingly low.

Perhaps the severity of the case illustrates why this is the first reported case of biallelic mutations in SHQ1, as such variants are not normally compatible with life. Unfortunately, the severity of the case and limited patient access also prevented analysis of the telltale sign of DC, extremely short telomeres. We may have to wait for another rare case of SHQ1 variants to manifest among DC patients before this gene can be definitively added to the growing list of those affected in DC.

## Conflict of Interest

The authors declare no conflict of interest.
